# Employing of Trukhan Model to Estimate Ion Transport Parameters in PVA Based Solid Polymer Electrolyte

**DOI:** 10.3390/polym11101694

**Published:** 2019-10-16

**Authors:** Shujahadeen B. Aziz, Rawezh B. Marif, M. A. Brza, M. H. Hamsan, M. F. Z. Kadir

**Affiliations:** 1 Advanced Polymeric Materials Research Lab., Department of Physics, College of Science, University of Sulaimani, Qlyasan Street, Sulaimani 46001, Kurdistan Regional Government, Iraq; rawezhmarif@gmail.com (R.B.M.); mohamad.brza@gmail.com (M.A.B.); 2 Komar Research Center (KRC), Komar University of Science and Technology, Sulaimani 46001, Kurdistan Regional Government, Iraq; 3 Manufacturing and Material Engineering, Faculty of Engineering, International Islamic University of Malaysia, Kuala Lumpur, Gombak 53100, Malaysia; 4 Centre for Foundation Studies in Science, University of Malaya, Kuala Lumpur 50603, Malaysia; hafizhamsan93@gmail.com (M.H.H.); mfzkadir@um.edu.my (M.F.Z.K.)

**Keywords:** solid polymer electrolyte, electrical impedance study, electrical equivalent circuits, bode plots, trukhan model, dielectric relaxation study, electric modulus study, ion transport mechanism

## Abstract

In the current paper, ion transport parameters in poly (vinyl alcohol) (PVA) based solid polymer electrolyte were examined using Trukhan model successfully. The desired amount of lithium trifluoromethanesulfonate (LiCF_3_SO_3_) was dissolved in PVA host polymer to synthesis of solid polymer electrolytes (SPEs). Ion transport parameters such as mobility (μ), diffusion coefficient (*D*), and charge carrier number density (*n*) are investigated in detail using impedance spectroscopy. The data results from impedance plots illustrated a decrement of bulk resistance with an increase in temperature. Using electrical equivalent circuits (EEC), electrical impedance plots (ZivsZr) are fitted at various temperatures. The results of impedance study demonstrated that the resistivity of the sample decreases with increasing temperature. The decrease of resistance or impedance with increasing temperature distinguished from Bode plots. The dielectric constant and dielectric loss values increased with an increase in temperature. The loss tangent peaks shifted to higher frequency region and the intensity increased with an increase in temperature. In this contribution, ion transport as a complicated subject in polymer physics is studied. The conductivity versus reciprocal of temperature was found to obey Arrhenius behavior type. The ion transport mechanism is discussed from the tanδ spectra. The ion transport parameters at ambient temperature are found to be 9 × 10^−8^ cm^2^/s, 0.8 × 10^17^ cm^−3^, and 3 × 10^−6^ cm^2^/Vs for *D*, *n*, andμ respectively. All these parameters have shown increasing as temperature increased. The electric modulus parameters are studied in an attempt to understand the relaxation dynamics and to clarify the relaxation process and ion dynamics relationship.

## 1. Introduction

Human life and earth planet have been threatened by current types of energy forms, so that researchers required thinking about a promising alternative which can be seen in electrical energy form [1]. In this regard, lithium-ion batteries are considered as one of popular source of electrical energy sources. For these to be applicable in a large scale, it needs for proper electrolytes which can be seen in solid polymer electrolytes (SPEs) have long been of interest to a number of researcher groups [2]. For example, polyethylene oxide (PEO) based polymer electrolytes are the most intensively studied [3], and found to be one of the most promising materials for Li-ion batteries [2,3]. Several properties make them to be appropriate electrolytes, such as plausible mechanical flexibility, cheapness [4,5]. On the other hand, relatively low ionic conductivity is one of the obvious drawbacks of PEO based polymer electrolytes [5]. Another example of polymer host of interest to many researcher groups is poly (vinyl alcohol) (PVA) [6,7]. It owes inherent properties, for instance reasonable potential host polymer, relatively high tensile strength, mechanical strength, thermal stability, dielectric constant and charge storage capacity [3,8]. The PVA based polymer electrolytes have shown suitability for applications in a many of electrochemical cells [9,10] and supercapacitors [11]. Dealing with the ionic conductivity of polymer electrolytes is absolutely impressive because of structure dependent characters, thereby, one can alter the structure of the material which in turn appropriate for specific task applications in an attempt to improve both chemical and physical properties [12,13].

There are three outstanding factors that estimate ionic conductivity in polymer electrolytes, such as ion mobility, concentration, and diffusion coefficient [14]. These are under intense exploration in PEO and PVA based polymer electrolytes [15,16,17,18,19]. There are numerous methods that used in measuring these quantities. The most recent and common one is ac impedance spectroscopy [6,20], whereas NMR spectroscopy can also be used to investigate mobility and relaxation behavior in polymer electrolytes [21]. The core concept for applying impedance spectroscopy has been employed to estimate the important transport parameters in polymer electrolytes [22]. Munar et al., [23], investigated a dielectric spectroscopy-based approach for determining the parameters, such as ionic mobility, concentration, and diffusion coefficient in lithium salts doped to polymer electrolytes. It is obviously realized that ion transport mechanism is not entirely comprehended in polymer physics and the numerous of information about ion conductivity motivates researchers to tackle with ion conducting polymer electrolytes [24,25,26,27,28]. The knowledge of the physical and chemical properties of polymeric materials from the molecular level to solid-state chemistry enables researchers to understand organic, coordination, and solid-state chemistry, catalysis, physics, materials science, and solid state chemistry and also to analyze electrical and mechanical properties of certain materials [27,29]. 

In this contribution, ion transport parameters for PVA:LiCF_3_SO_3_ solid electrolyte has been explored using the Trukhan model. In this model, it is allowed to use the values related with *f_max_* and *tan(δ)_max_* in loss tangent plots and to calculate the ion transport parameters, such as mobility (μ), diffusion coefficient (*D*), and charge carrier number density (*n*). Once the values of the diffusion coefficient are attained, it is also easy to calculate mobility (μ) and charge carrier number density (*n*) from both diffusion coefficients and DC conductivity at various temperatures.

## 2. Experimental Method

### 2.1. Materials and Sample Preparation

Sigma Aldrich (Kuala Lumpur, Malaysia) supplied the Poly (vinyl alcohol) (PVA) (averageMw85,000–124,000,87%–89%) powder material. In the present work, solid polymer electrolytes based on PVA were synthesised by a facile conventional solution cast technique. The procedure includes dissolution of one gram of PVA in 50 mL of distilled water at 90 °C. The solution was stirred continuously with aid of magnetic stirrer for several hours until the PVA powder was completely dissolved, to obtain homogeneous viscous solution. Afterwards, the PVA solution was left to cool down to room temperature. Subsequently, 10 wt.% of lithium trifluoromethanesulfonate (LiCF_3_SO_3_) (Sigma-Aldrich, Kuala Lumpur, Malaysia) [CAS Number 33454-82-9, Molecular Weight = 156.01 g/mol] was added to the solution to make it alkaline solution of PVA:LiCF_3_SO_3_ polymer electrolyte. Then, the mixture was stirred continuously until a homogeneous solution was obtained. Ultimately, after casting in Petri dish (90 mm × 15 mm, Sigma-Aldrich, Kuala Lumpur, Malaysia), the solution was left to dry to form a film at room temperature. The films produced were then put into desiccators for extra drying and moisture elimination.

### 2.2. Impedance Measurement

The impedance spectra of the films was measured using HIOKI 3531 Z Hi-tester (No. 1036555, Hioki, Nagano, Japan) in the frequency range of 50 Hz to 1000 kHz and at various temperature ranging from 303 K to 353 K. The tester was connected to a computer and software recorded both the real and imaginary parts of impedance spectra. The SPE films were cut into small discs of 2 cm in diameter and placed between two stainless steel electrodes under spring pressure. From the analysis of the real (*Z*r) and imaginary (*Z*i) parts of complex impedance (Z*) spectra, complex permittivity (ε*) and complex electric modulus (M*) can be extracted.

## 3. Results and Discussion

### 3.1. Complex Impedance Analysis

**Figure 1:**
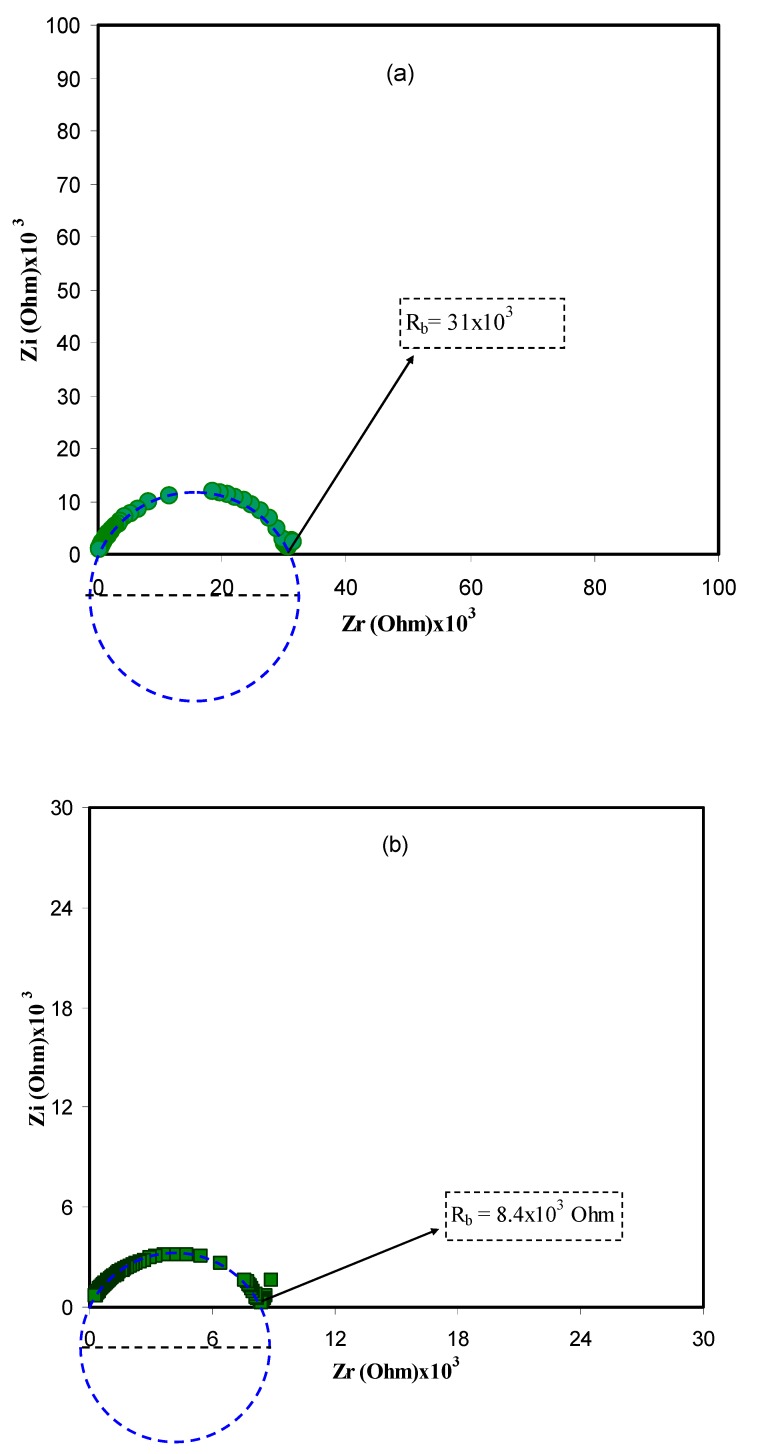

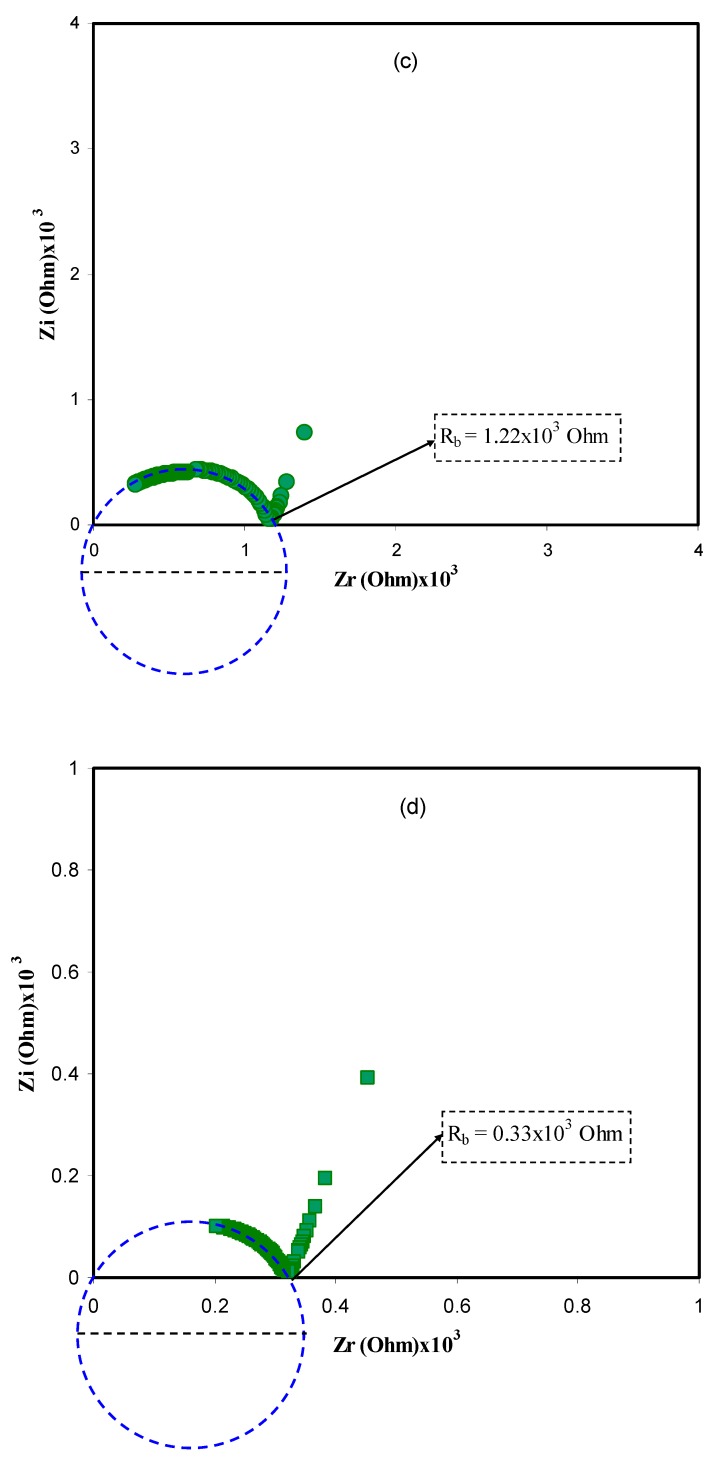
Complex impedance plot of PVA:LiFC_3_SO_3_ (*Z*ivs*Z*r) solid electrolyte at (**a**) 303 K, (**b**) 323 K (**c**) 343 K, and (**d**) 353 K. The insets indicate the corresponding bulk resistance *R*_b_. Clearly from 303 K to 353 K the diameter of the semicircles in impedance plots decreases and the spike regions were increased.

Complex impedance spectroscopy (CIS) is a powerful technique applied to study the mechanism of both ion transport and charge transfer in electrolytes and at electrodes, respectively at various frequencies. The technique is relatively straightforward and informative in the studying electrochemical behavior of electrodes and ion transport of ion conducting materials including polymer electrolytes [30,31]. Herein, the technique is also used in analyzing electrical impedance plots (*Z*ivs*Z*r) for all the samples at various temperatures as shown in Figure 1a–d. One can see a characteristic small arc, with a center that is well below the real axis, at the high frequencies, and a linear tail at the low frequencies. The plot can be modeled by an equivalent circuit consisting of a capacitor and a resistor corresponds to immobile polymer chains and movement of ions, respectively as can be seen in later sections [32]. The spike at the low frequency region was resulted from electric double layer (EDL) capacitances formation by the free charge accumulation at the interfacial region of the solid electrolyte and electrode surface [33]. In fact, the migration of ions and the surface heterogeneity of the blocking electrodes are responsible for the inclination of data points at the low frequency [34]. The semi-circles shrinking with an increase in temperature, indicating low resistance of the sample since the bulk resistance (*R*_b_) is reflected in intercept of the arc with the *Z*raxis. This increase in conductivity is posed by an increase of both charge carrier mobility and carrier density. The nearly disappearance of the arc in the final plot (Figure 1d) means dominating ionic conduction of the whole conductivity [35]. The intercept of the arc with the real axis (*Z*r) at the low frequency (end) made the bulk (ionic) resistance (*R*_b_) within the materials [36].

**Figure 2:**
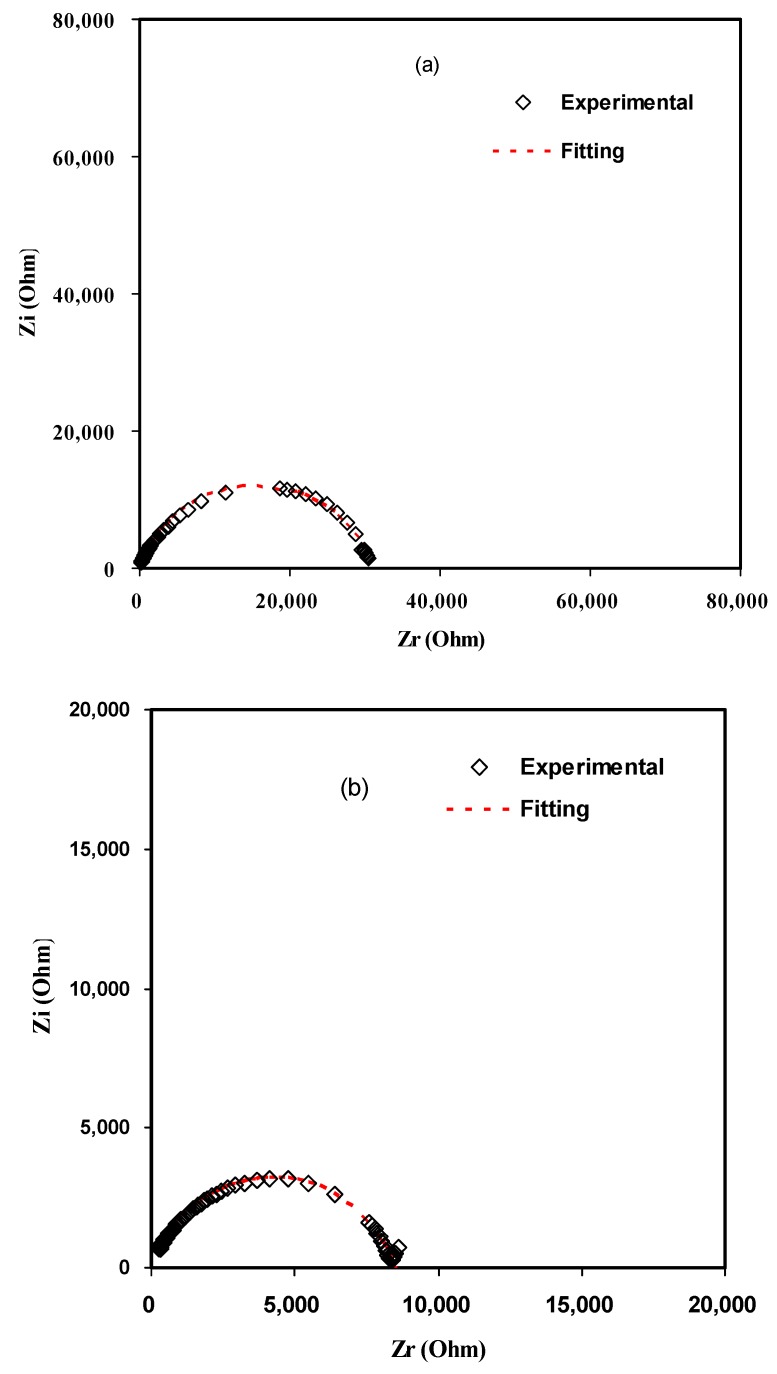

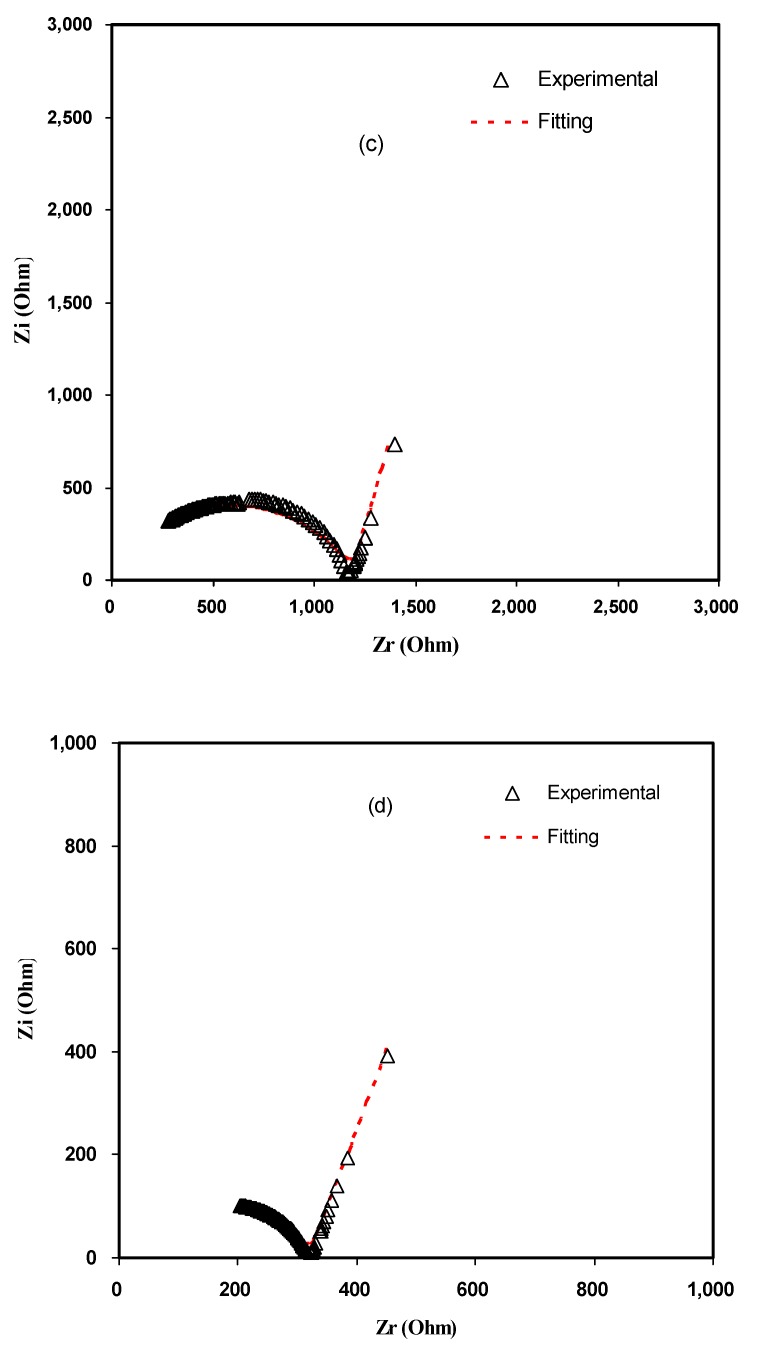
Experimental and fitting (EEC) Impedance (Nyquist) plots at (**a**) 303 K, (**b**) 323 K, (**c**) 343 K, and (**d**) 353 K.

**Figure 3:**
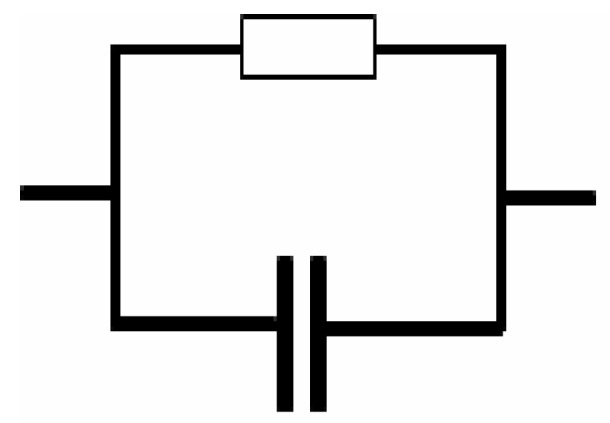
Schematic illustration of the electrical equivalent circuit (EEC). Resistor is represented by the symbol 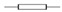 and capacitor represented by 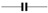.

For the sake of clarity, the impedance plots in Figure 1 are simulated with electrical equivalent circuits (EECs). Generally, modeling and investigation of the spectra of impedance are facilitated with an equivalent circuit, which stemmed from the fact that it is an easy and provides the mechanism of the system [37]. Figure 2a–d illustrates the experimental impedance plots with EECs for selected samples. The EEC model is essential to grasp the electrical properties of polymer based solid electrolytes. It is feasible to represent experimental impedance plots using a three-component EEC. More precisely, the three chief components are *Z*_CPE1_, a constant phase element; *Z*_CPE2_, another constant phase element; and a bulk resistance (*R_b_*) for the SPE. *R_b_* and *Z*_CPE1_ are reflected in the high frequency region, while the low frequency spike region is linked to *Z*_CPE2_. This is indicative to the double layer capacitance emerging at the electrodes and the SPEs interface [38]. The common equation for constant phase element (CPE) is as follows [39,40,41]:

(1) $$Z_{\mathrm{CPE}}=\frac{1}{C\omega^{p}}\left[\cos\left(\frac{\pi p}{2}\right)-i\sin\left(\frac{\pi p}{2}\right)\right]$$

where *p* is related the deviation of the plot from the axis while *Z*i and *Z*r are indicators of imaginary and real parts of the impedance, respectively. C is the capacitance for the CPE and angular frequency is denoted as ω. In particular, CPE is acronym generally applied in place of capacitor in the context of a modeled EEC. This is due to the fact that the behavior of SPEs is different from the behavior of an ideal or pure capacitor. This denotes an ideal semi-circular pattern [38], which it is not likely to identify in existing experimental impedance plots. In this case, it is probable to express the real (*Z*r) and imaginary (*Z*i) complex impedance (Z*) values in the EECs for semicircle regions in the following way:

(2) $$Z_{r}=\frac{R_{b}C\omega^{p}\cos\left(\frac{\pi p}{2}\right)+R_{b}}{2R_{b}C\omega^{p}\cos\left(\frac{\pi p}{2}\right)+R_{b}{}^{2}C^{2}\omega^{2p}+1}$$

(3) $$Z_{i}=\frac{R_{b}{}^{2}C\omega^{p}\cos\left(\frac{\pi p}{2}\right)}{R_{b}C\omega^{p}\cos\left(\frac{\pi p}{2}\right)+R_{b}{}^{2}C^{2}\omega^{2p}+1}$$

here *R*_b_ is the bulk resistance which are obtained from the intersects of Figure 1. Based on Equations (2) and (3), Figure 2a,b is simulated and little data at low frequency are neglected. Schematically the EEC corresponding to Figure 2a,b is shown in Figure 3. However, for Figure 2c,d many data at low frequencies can be distinguished and thus the low frequency regions cannot further be neglected. The equations for impedance plots consists of semicircle (high frequency region) and spike (low frequency tail) areas follows:

(4) $$Z_{r}=\frac{R_{b}C_{1}\omega^{p1}\cos\left(\frac{\pi p_{1}}{2}\right)+R_{b}}{2R_{b}C_{1}\omega^{p}\cos\left(\frac{\pi p}{2}\right)+R_{b}{}^{2}C^{2}\omega^{2p}+1}+\frac{\cos\left(\frac{\pi p_{2}}{2}\right)}{C_{2}\omega^{p2}}$$

(5) $$Z_{i}=\frac{R_{b}C_{1}\omega^{p1}\sin\left(\frac{\pi p_{1}}{2}\right)}{2R_{b}C_{1}\omega^{p}\cos\left(\frac{\pi p}{2}\right)+R_{b}{}^{2}C^{2}\omega^{2p}+1}+\frac{\sin\left(\frac{\pi p_{2}}{2}\right)}{C_{2}\omega^{p2}}$$

**Figure 4:**
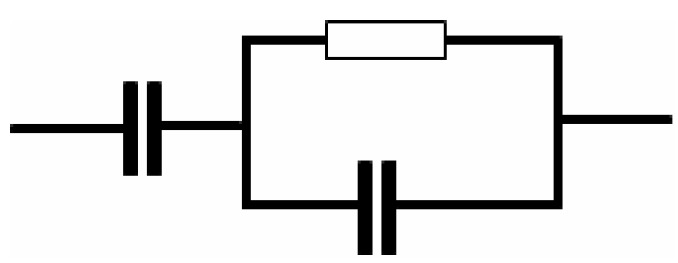
Schematic illustration of the electrical equivalent circuit (EEC). Resistor is represented by the symbol 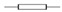 and capacitor represented by 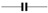.

Based on Equations (4) and (5) the experimental impedance plots at high temperatures are well simulated. It is clear that the diameter of the semicircle shrunk at the high frequency region under an increasing of temperature. Figure 2c,d shows that the incomplete semicircle corresponds to a parallel combination of *R*_b_ with CPE element and in series with another CPE corresponding to low frequency tail as shown schematically in Figure 4. Table 1 tabulates all the parameters accomplished by fit for the plots of impedance with equivalent circuits. 

**Figure 5:**
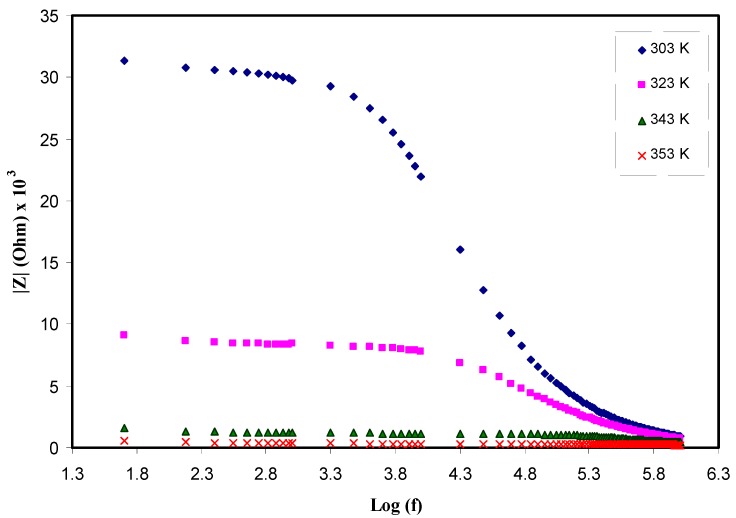
Bodeplot (experimental and fitting) for PVA:LiCF_3_SO_3_ at various temperatures.

Moreover, the above elucidation and proposed semicircles for Nyquist plots are further supported by investigating Bode plots. Figure 5 shows the Bode plots for the electrolyte samples at room temperature. It has been demonstrated from the earlier studies that the Bode plots should exhibit three discriminated regions: capacitive (plateau at low frequency), diffusion (inclined region), and charge transfer regions (high frequency) [42,43]. Clearly three regions can be seen in the Bode plot. The plateau region at low frequency is ascribed to capacitive behavior while, the intermediate frequency region attributed to the diffusion and charge transfer regions is appeared at high frequency plateau region. As demonstrated in impedance plots (see Figure 1), the semicircle is associated to transport of ions in amorphous phase of electrolytes and the tails are referred to diffusion contribution. Obviously, with rising temperature, the diffusion contribution is raised and the resistance declined, due to the increase of mobility of ions and concentration of carriers at elevated temperatures as can be observed in later sections. Normally, the spikes at low frequency regions are signified by CPE, which are in series with equivalent circuit elements. Other researchers have also used EECs to illustrate the Bode and impedance plots [44,45]. Clearly with rising temperature as shown in Figure 5 the charge transfer resistance dropped. The low frequency dispersion region in the Bode plots is attributed to the phenomena of ion diffusion while the high frequency region is ascribed to the charge transfer resistance [46]. Hence, the Bode plots also supported the results obtained from the impedance plots. From the viewpoint of physics it is important to provide polymer electrolytes with high DC conductivity and from the viewpoint of chemistry it is essential for the samples to have the low charge transfer resistance.

### 3.2. Dielectric Properties

**Figure 6:**
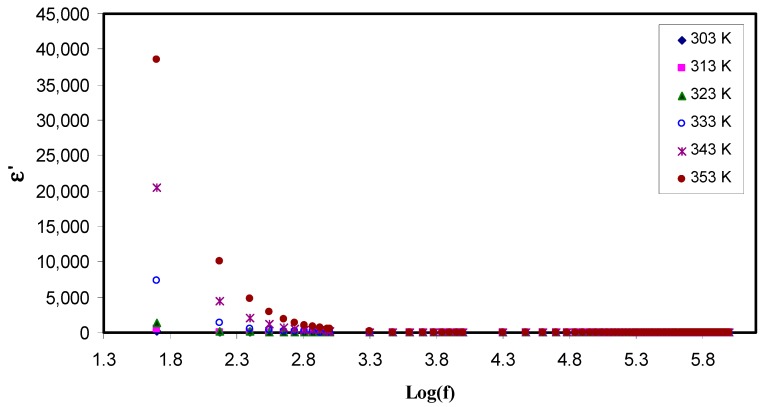
Constant(ε′) as a function of the logarithm of frequency (*f*) at different temperatures. It is obvious that the dielectric constant increases with increasing temperature at low frequency region due to electrode polarization effect.

**Figure 7:**
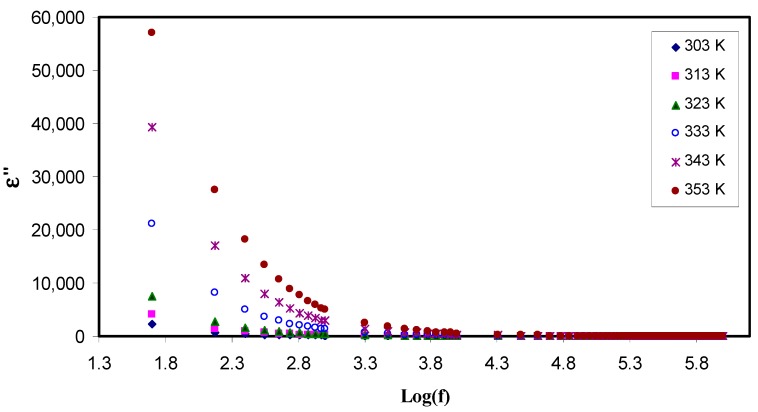
Dielectric loss (ε″) as a function of the logarithm of frequency (*f*) at different temperatures. The dispersion in dielectric loss spectra rises with rising temperature.

To examine the ionic conductivity of polymer electrolyte, it is obeyed that the dielectric properties has to be measured. In an attempt to improve conductivity, the number of free mobile ions in the polymer electrolyte is increased [47]. These properties provide an insight into both the conductivity and the crystallinity of polymers [48]. For this to perform, the dielectric spectroscopic technique allows one to investigate the various dynamic mechanisms in electrolytes. Its principle is based on perturbation by applying an alternating field into the electrolyte at different frequencies, and recording the response that reflected in dielectric constant (ε) measurement. The two parts of complex dielectric function (ε*) are the real (ε′) and the imaginary (ε″) which correspond to energy stored in the material, and dissipated energy, respectively. Figure 6 and Figure 7 show ε′ and ε″ as a function of frequency at various temperatures. From these two graphs, one can notice two obvious regions. One region is located at the low frequency where the permittivity is very relatively quite large, as a result of the accumulation of charge at the electrode-electrolyte interface which is called the electrode polarization (EP). The second region is appeared at the high frequency where molecular dipoles cannot stay constant with the rapidly changing field. One more observation is at the tail end of graph, the ε′ and ε″ graph become independent of frequency, i.e., remain constant. It can be interpreted on the basis of that there is a lack of excess ion diffusion parallel to the field when the change in polarity of the field met fast enough, i.e., when the frequency is relatively very high [49]. A general trend observed in both figures is an increase in permittivity with increases in temperature. This is resulted from the fact as the temperature increased; it helps facilitation of dipole orientation. It also reinforces charge carrier density as a consequence of increasing salt dissociation and dissolution. These behaviors are common to occur in some polymer electrolytes as documented in the literature [49,50,51]. To look at this phenomenon in deep, there is the existence of forces in polymer materials which generally classified into primary (intra-chain) and secondary (inter-chain) forces which in turn stabilize the polymer structure [52]. The primary forces arise from the covalent bond formation (2.2–8.6 eV) to bind the chains of backbone atoms together. However, there are four different secondary forces in polymers, which are dipole-dipole bonding (0.43–0.87 eV), hydrogen bonding (0.13–0.30 eV), induced interaction (0.07–0.13 eV) and dispersion interaction (0.002–0.09 eV). These forces possess relatively low dissociation energies; thereby, these forces are susceptible to temperature change than their primary counterpart. This behavior of temperature dependency of these forces causes the nature and extent of molecular motions in polymer is flexible, which in turn, impact their dielectric behavior, charge transport and charge storage properties. It is self-evident as temperature increases, the degree of salt dissociation and re-dissociation of salts increase, showing an increase in the number of free ions or charge carrier density [53].

It is well-defined that both dielectric constant (ε′) and number density of charge carriers (*n_i_*) are strongly are interrelated through relation,$n_{i}=n_{o}\;\exp\;(-U/\varepsilon^{\prime }K_{B}T)$, where U is the dissociation energy, T is the absolute temperature, *n*_0_ is a pre-exponential constant, and K_B_ is the Boltzmann’s constant. Likewise, an increase in dielectric constant means the increase in DC conductivity. It is self-evident that the DC ionic conductivity of polymer ion-conducting electrolytes depends upon both the charge density (*n_i_*) and the mobility (µ_i_) (σ = *Σ qn*_i_µ_i_), where q is the charge on ion carriers [24,25,28]. Therefore, examining the dielectric constant can be an informative parameter and leading to a deep understanding the electrical properties of polymer electrolytes and consequently predicting the conductivity behaviors of the samples. From the Figure 7, one can observe that the dielectric loss is relatively large compared to the dielectric constant (see Figure 6) at the lower frequencies, this may result from a huge difference in the free charge motion in the material [35]. It was also found that dielectric relaxation primarily resulted from reorientation process of dipoles in the polymer chains, and as a consequence it shows a peak in the ε″ spectra. However, the ion cooperative motions cause the relaxation peaks to be hidden in the ε″ spectra [54]. 

### 3.3. Electric Modulus Analysis

**Figure 8:**
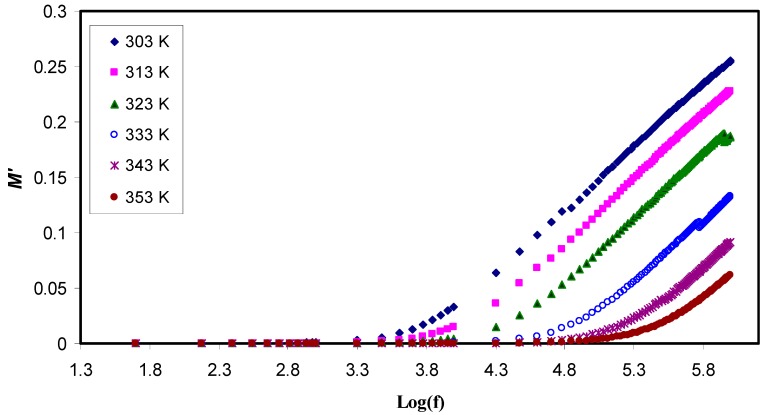
Realpart of the electric modulus (*M*′) as a function of the logarithm of frequency (*f*) at different temperatures. It is obvious that M′ decreases with increasing temperature at high frequency region while it has a low value at the low frequency region due to the suppression of the electrode polarization effect.

**Figure 9:**
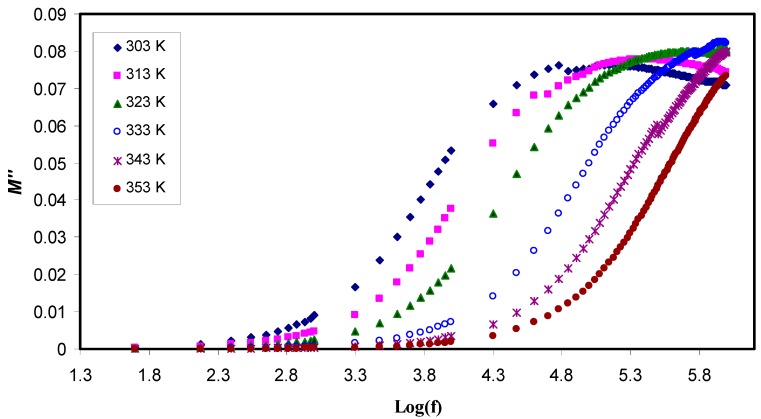
Imaginary part of the electric modulus (*M*″) as a function of the logarithm of frequency (*f*) at different temperatures. It is obvious that M″ decreases with increasing temperature at high frequency region while it has a low value at the low frequency region due to the suppression of the electrode polarization.

From electric modulus analysis, one can deliver insights into relaxation processes. This is owing to the inverse of the permittivity is considered in electric modulus and hence the great value of the permittivity because of electrode polarization (EP) effects at the low frequency can be suppressed [55]. The main difficulties can be overcome which prevented analysis comprehensively and descriptively relaxation in permittivity such as electrode nature, space charge phenomena and conduction effects [56,57,58,59,60,61]. Recently, a number of studies revealed that conductivity and relaxation dynamic of polymer electrolytes depend mainly upon the frequency and they are also strongly susceptible to the motion of charge species and dipoles of the polymers [58,59,60,61]. Implementation of electric modulus formalism enables investigation and analysis of the dielectric relaxation [57]. It is applicable to use the real (*Z*r) and imaginary (*Z*i) parts of complex impedance (Z*) in computing the real and imaginary parts of complex electric modulus (M*), using the following formulae [57,58,59]:

(6) $$M^{\prime }=\frac{\varepsilon^{\prime }}{{(\varepsilon }^{\prime 2}{+\varepsilon }^{\prime \prime 2})}=\omega C_{o}Z^{\prime \prime }$$

(7) $$M^{\prime \prime }=\frac{\varepsilon^{\prime \prime }}{{(\varepsilon }^{\prime 2}{+\varepsilon }^{\prime \prime 2})}=\omega C_{o}Z^{\prime }$$

where ω is the angular frequency, C_o_ is the capacitance of dielectric cell without the sample, *Z*r is the real part of impedance and *Z*i is the imaginary part of the impedance. Figure 8 and Figure 9 exhibit both the real and the imaginary part of the electric modulus, *M*′ and *M*″, respectively. On the one hand, it can be observed that both *M*′ and *M*″ have a low value at the low frequency region, confirming the suppression of the EP effect. On the other hand, at the higher frequencies, both parts of the electric modulus increase and peaks can be noticed clearly in some of the *M*″ lines while some others tend to go beyond the experimental capabilities. All these are considered the conduction relaxation peaks. Below the peak frequencies (f_max_)the charge carries are mobile over a long distance [55].

The study of effect of temperature has showed as temperature increases, decrease M′ and a shift of the *M*″ peaks to the higher frequencies occurred. This is because at the higher temperatures, the charge carriers are more mobile [62]. At low frequencies, there is an inclination of M′ values that can be attributed to the large capacitance associated with solid electrolytes. Figure 8 shows that *M*′ also decreases with temperature increasing as a consequence of an increase in the mobility of the polymer segments and charge carriers, in contrast, the permittivity (ε′) increases. Accordingly, both *Z*r and *Z*i decrease while conductivity of polymer electrolyte noticeably grows, indicating a strong coupling between ionic movement and the polymer segmental motion exhibited obvious peak in the M″ spectra [63] and no corresponding characteristics in the ε″ spectra (see Figure 7). These peaks’ appearance came from the charge carriers that confined within potential wells and being mobile over a short distance at the higher frequencies, in the other words, the peaks could be regarded as two extremes from the transition regions from long-range ionic mobility (translation) to short-range mobility (dipolar) [64]. Moreover, huge peak appearance is associated with the M″ spectra shifting forward with temperature, suggesting that as temperature increases, the conductivity relaxation time decreases simultaneously.

It is noting that there are no features associated with Debye type behavior confirming that the total conductivity may result from the migration of free ions together with both viscoelastic and dipolar relaxations [65]. Two features; broadness and asymmetry of the shapes of electric modulus (*M*″) in these plots are generally represented by the extended exponential decay function of the electric field [66] as shown in Equation (8)

(8) $$\varphi =\exp\left[{\left(-\frac{t}{\tau }\right)}^{\beta }\right],\;0<\beta <1,$$

**Figure 10:**
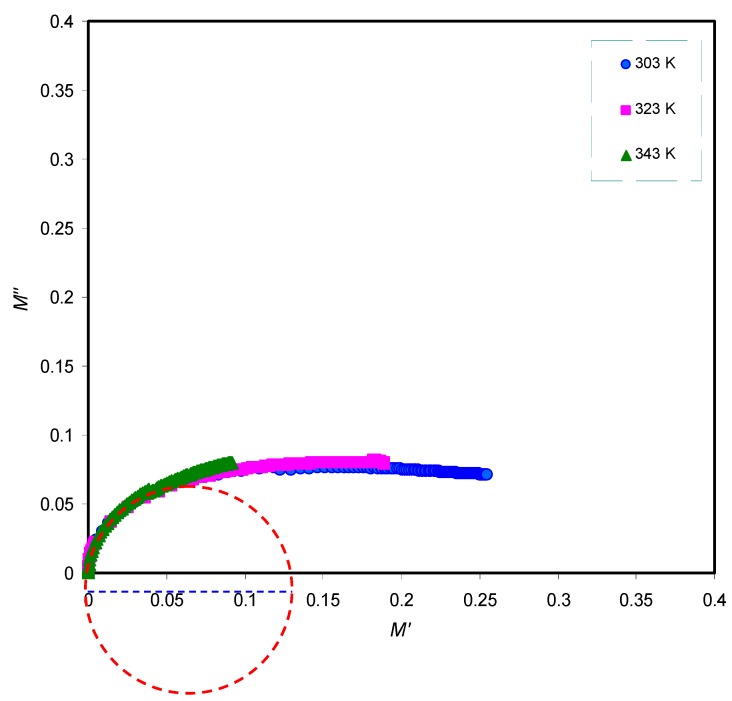
Argand plot at selected temperatures for PVA:LiFC_3_SO_3_solid electrolyte. The incomplete semicircles indicate the distribution of relaxation times.

The stretching parameter β is equivalent to 1.14/w, where w is full-width at half-maximum (FWHM) and it is 1.14 for Debye relaxation. In order to realize the relaxation dynamic mechanism in polymer electrolyte via viscoelastic relaxation or ionic conductivity relaxation, it is necessary to look at the Argand plots at various temperatures [66]. The Argand plot is presented in Figure 10 at selected temperatures. It is clear that the semicircles are incomplete and the diameter well below the real axis which indicates the distribution of relaxation times.

### 3.4. DC Conductivity Analysis 

**Figure 11:**
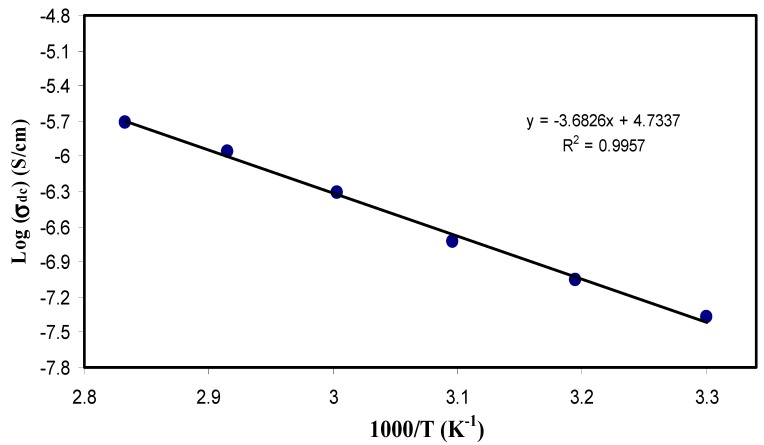
Logarithm of DC conductivity (σ_dc_) as a function of the inverse of temperature (1000/T). The linear behavior of DC conductivity versus the reciprocal of temperature reveals that ion transport follows the Arrhenius model.

Temperature study of DC conductivity (σ_dc_) has shown impressive information about the Arrhenius-like behavior of the polymer electrolyte. From the impedance plots, the bulk resistance (*R*_b_) was determined at various temperatures. The thickness of the sample (*l*) and its effective area (*A*) were used to calculate the DC conductivity at various temperatures (σ_dc_ = l/*R*_b_A). Figure 11 shows plot of the log σ_dc_ as a function of 10^3^ T^−1^. From the graph, it seems that the conductivity behaves linearly when plotted against the inverse of temperature. Arrhenius conductivity is given by:

(9) $$\sigma_{\mathrm{dc}}=\sigma_{o}e^{\frac{-E_{a}}{K_{B}T}}$$

where *E*_a_ is the activation energy, *K*_B_ is the Boltzmann constant and σ_o_ is pre-exponential factor.Thus, it is confirmed that the polymer electrolyte behaves in good agreement with the Arrhenius equation. This behavior is in accordance with the ones obtained from literature [18,67,68]. This segmental motion of the polymer chain causes this increase in conductivity with the temperature elevation. To pinpoint this, there is a mechanistic effect of ionic motion free volume of the whole system, allowing hoping of ions through sites, in the other words, it provides pathways for ions to move through quickly. More clearly, it means that the ionic motion is translational and mediated by the segmental motion. Interestingly, temperature elevation makes the polymer more amorphous (disordered), allowing for faster internal modes in the polymer chain, the bond rotations in these modes produce segmental motion that favors intra- and inter-chain ion hopping, as a consequence, conductivity increased [6,69].

### 3.5. Loss Tangent Analysis and Ion Transport Parameters

#### 3.5.1. Loss Tangent Analysis

Herein, there is a chance to extract more information about the relaxation dynamics from tanδ plots against frequency at various temperatures. The ionic conduction mechanism in the SPE materials is still not fully understood because of the coexistence of both crystalline and amorphous phases. In a complementary study, it is therefore important to understand the mechanism of ion transport deeply accompanying with the processes of polymer segmental relaxation in polymer electrolytes [24,25,58,70,71,72]. The mechanism of ion transport is still controversial among many researcher groups that work in this area. 

**Figure 12:**
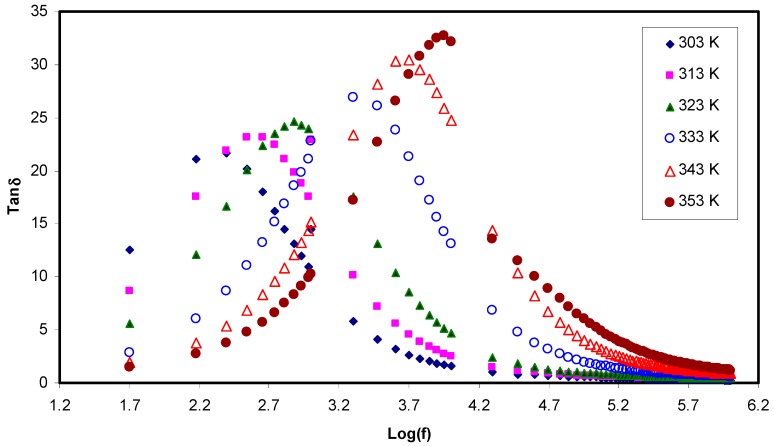
Tanδ as a function of the logarithm of frequency (f) at different temperatures. Tanδ increases with increasing frequency till reaching a maximum value at different temperatures and followed by it decreases with an increase in frequency.

Dielectric loss tangent is expressed as the ratio of ε″ to ε′ ($\tan\left(\delta \right)=\frac{\varepsilon^{\prime \prime }}{\varepsilon^{\prime }}$). Figure 12 shows the loss tangent against the logarithm of frequency for PVA:LiCF_3_SO_3_ at various temperatures. The loss tangent increases with frequency up till a peak is maximized; afterwards, it decreases with an increase in frequency. This is largely understood from how the resistive (ε″) and the capacitive (ε′) elements of the electrolyte respond to this alteration in frequency. At the lower frequency, the capacitive element is modeled as an open circuit, so the resistive element contributes increasingly, whereas at the high frequency, the capacitive element dominates remarkably [49]. From Figure 12, it seems that the position and the height of the peak as well increase with an increase in temperature. This is explained in terms of the fact that the higher temperature enables charge carrier movement to be easier and thus capable of reaching relaxation at the higher frequency [19]. This finding is of significant importance in which these tangent loss peaks and shifts with the temperature provide insight into the dielectric relaxation process that is thermally activated in the samples [73,74]. Comparably, the ion hopping from one site to another is as in crystalline ionic materials. 

Meaningful view, in polymer electrolytes with plausible electrical conductivity, dielectric relaxation peaks owing to permanent or induced dipoles may be prohibited by the relaxation from polarization of mobile charged species exists in the material. The low frequency relaxation peaks disappeared in the current work is related to the coupling of relaxation peaks with carrier motions [75]. The tanδ shape in Figure 12 can be interpreted in terms of model of Koops phenomenology [76]. Accordingly, loss tangent increases with an increase in the frequency, showing a maximum at a particular frequency at various temperatures. This belongs to the ohmic component of current increases more rapidly than its capacitive component part. It is apparent that the capacitive component (Zi = 1/2πfC) is relatively very small at the high frequency region. The fitting of impedance plots with EECs exactly supports our interpretation to tanδ plot. In contrast, at the higher frequency region, the loss tangent decreases with an increase in the frequency because the ohmic component of current is virtually frequency independent. As a result, the large value of the frequency (f) makes the capacitive component to be increased in proportion to frequency [77,78]. The broadness feature of the loss tangent peak emphasizes that the relaxation process is non-Debye relaxation and confirmed from the electric modulus analysis as well. 

Paramount importance finding is the increase in height of tanδ with temperature could be correlated to a decrease in resistivity of the whole sample [79]. Recent advance made in the study of loss tangent peaks at various temperatures are powerful to probe the relaxation peaks such as α, β and γ relaxations. These are attributed to dipole rotation in crystalline phase, dipole orientation in amorphous regions correspond to the movement of side groups or end-groups in the amorphous phase, respectively [60]. In this contribution, it is understood that the shape and intensity of tanδ peaks at various temperatures are completely dependent on the ion mobility and diffusivity. The values of tan (δ)_max_ and frequency can be used to plot mobility (μ), carrier density (*n*) and diffusivity (*D*) as a function of temperature, which will be at the top of discussion in the next sections.

#### 3.5.2. Diffusion Coefficient Analysis

**Figure 13:**
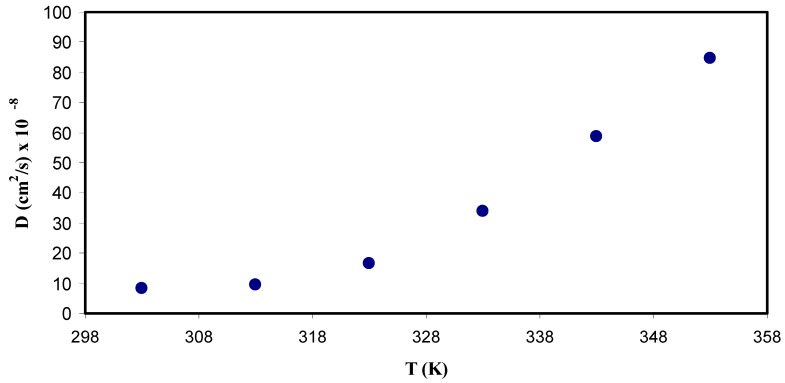
Diffusion coefficient (*D*) as a function of temperature (*T*). It is obvious that diffusion coefficient of ions increases nonlinearly with temperature.

It is well assumed to say that ion conducting electrolyte is heart of all electrochemical devices. Prior to use in electrochemical applications, such as battery and supercapacitor, the electrical properties electrolyte have to be characterized. Among these properties, DC conductivity has to be well analyzed [39]. From analysis of loss tangent, the tanδ plots are correlated to both the capacitive and resistive component of the solid electrolytes. Furthermore, it is explained that this shift of tanδ peaks towards the high frequency region is associated to the ions that thermally activated as well as the DC conductivity pattern versus 1000/T supported this interpretation for tanδ peak shifts. In an attempt, to calculate three properties, such as the number density, mobility and diffusion coefficient of the charge carriers, the Trukhan model has been employed to the loss tangent data results. In this model, diffusion coefficient of cations and anions are supposed to be the same, so a simple expression can be used to calculate the diffusion coefficient from the peaks appeared in the plots of loss tangent versus frequency. The expression is as follows:

(10) $$D=\frac{2\;\pi \;f_{\max}\;L^{2}}{32\tan^{3}(\delta {)}_{\max}}$$

where *L* is the sample thickness [23]. Figure 13 shows a graph between the diffusion coefficient and the temperature. It can be clearly seen that the diffusion increases with the temperature non-linearly. This randomness of the data points might be mainly due to that diffusion is facilitated by segmental motion rather than pure ionic motion [80], and partly the increase in temperature allowed for more favorable modes of segmental motion. One more interesting observation is that the diffusion parameter measured to be 9 × 10^−8^ cm^2^/s and 9.2 × 10^−7^ cm^2^/s at the room temperature and 353 K, respectively which by one order of magnitude. The value of diffusion coefficient obtained in the present work is quite comparable to that reported for PEO based ion conducting polymer electrolytes using Nernst−Einstein equation [81]. From Nernst−Einstein, one can convert the conductivity into a diffusion coefficient and vice versa, i.e., the charge diffusivity D_σ_ can be expressed as:

(11) $$D_{\sigma }=\frac{K_{B}T}{C_{\mathrm{salt}}e^{2}}\sigma_{\mathrm{dc}}$$

where all parameters except *C*_salt_ in the relation have usual meanings. It is remarkable that Equation (11) contains the known salt concentration *C*_salt_ (number density of molecules) in place of the unknown concentration of free (dissociated) ions. In a comparison, the Trukhan is easy to be applied than the Nernst−Einstein because it is too complicate to predict the number of free ions and ion aggregates in polymer electrolytes. As far as we know, free ions, ion pair, ion multiple and ion aggregates exist in polymer electrolytes [82]. Arya and Sharma, recently studied the electrical conductivity value of particular SPEs films and they found that the conductivity depends on the number of free charge carriers. In their study, the obtained diffusion coefficient of about (≈10^−18^ cm^2^/s) for SPEs based on PEO-PVP complexed with NaPF_6_ [83] which is very low compared to the values obtained in the current work and that reported by other researchers [81]. Moreover, Sun et al., [84], computed the diffusion coefficient of almost (≈10^−10^ cm^2^/s) for poly (trimethylene carbonate) based Li ion conducting electrolyte.

#### 3.5.3. Carrier Density Study

**Figure 14:**
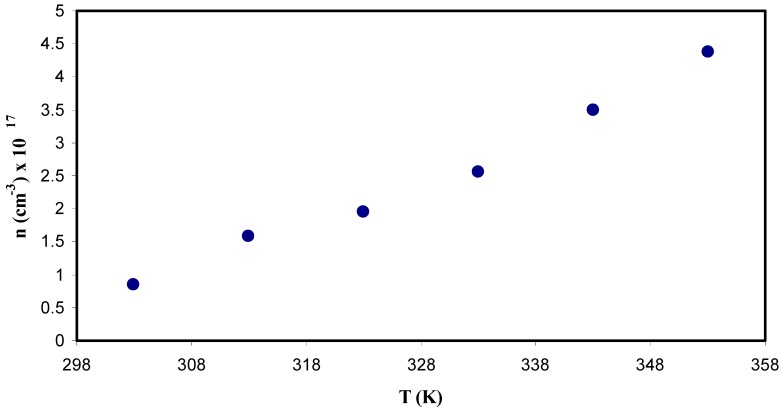
Carrier number density as a function of temperature. It is clear that the number density of ions slightly increases with increasing temperature.

Another important parameter is the number of density of mobile ions that correlated to the conductivity. The number density of mobile ions (n) can be estimated from the well-known Einstein relation as shown below:

(12) $$n=\frac{\sigma_{\mathrm{dc}}K_{B}T}{De^{2}}$$

where *σ*_dc_ is DC conductivity, K_B_ is the Boltzmann constant, T is the absolute temperature, D is the diffusion coefficient and e is the elementary charge. Figure 14 exhibits number density of mobile ions (n) as a function of the temperature. It is noticeable that there is a slight increase in n as temperature increases, which remains less than an order of magnitude. The logical explanation of influence of the temperature may be by considering the ions bound in the crystalline parts of the polymer matrix are released as a result of making the polymer more amorphous [23]. From the literature, a number of different models were reported in calculating the number density (n) of ions in polymer electrolytes. Among them, the Rice and Roth model [85] states that conductivity can be expressed as:

(13) $$\sigma =\frac{1}{3}\left[\frac{{\left(Ze\right)}^{2}}{K_{B}T}\right]nvl\exp\left(-\frac{E_{a}}{K_{B}T}\right)$$

It is seen from the above equation, to obtain the jump distance, *l*, between the transit sites or the distance between two coordinating sites must be known. And v is the velocity of the ionic carrier given by the following equation:

(14) $$v=\sqrt{\frac{2E_{a}}{M}}$$

where M is the mass of the ion and $E_{a}$ is the activation energy. The value of n for the polymer-salt system has been calculated by some researchers [86,87,88]. Maurya et al. [86] calculated n for the PEO-NH_4_CIO_4_ electrolyte system using transient ionic current technique and the n values were in the range of 10^6^ to 10^17^ cm^−3^. Winie et al. [89] computed the number density of mobile ions (10^17^ to 10^19^ cm^−3^) using the Rice and Roth equation for plasticized hexanoyl chitosan-lithium salt polymer electrolyte, and Majid and Arof [87], obtained the value of number density (n) in the range 10^18^ to 10^19^cm^−3^. Chandra et al. [88] documented the value of number density (n) in the range of 10^16^ to 10^18^ cm^−3^ for PEO:PVP:AgNO_3_ based SPEs. Agrawal et. al. [90] also reported the mobile of ion concentration in the range 10^15^ to 10^16^ cm^−3^ for hot press PEO:AgNO_3_:SiO_2_nano-composite system. It is also reported that the conductivity obtained is dependent on both the number of mobile ions and mobility [90]. In this contribution, the carrier density of 0.8 × 10^17^ cm^−3^ is comparable to those reported for polymer based solid electrolytes in the literature [86,87,88,89,90]. 

The present study aims at pinpointing that the Trukhan model is relatively an accurate method to calculate the transport parameters associated with the ion movements in polymer electrolytes. In a comparison, both Rice and Roth and Trukhan models are two important models, where it is clearly seen that the Rice and Roth model is a complicated model that depends upon several parameters which are difficult to estimate correctly, for instance, the jump distance (*l*) and activation energy (*E*_a_). Hence the Trukhan model has showed a deeper understanding in terms of number density and mobility of ions on variation of the conductivity than the other model.

#### 3.5.4. Ion Mobility Analysis

**Figure 15:**
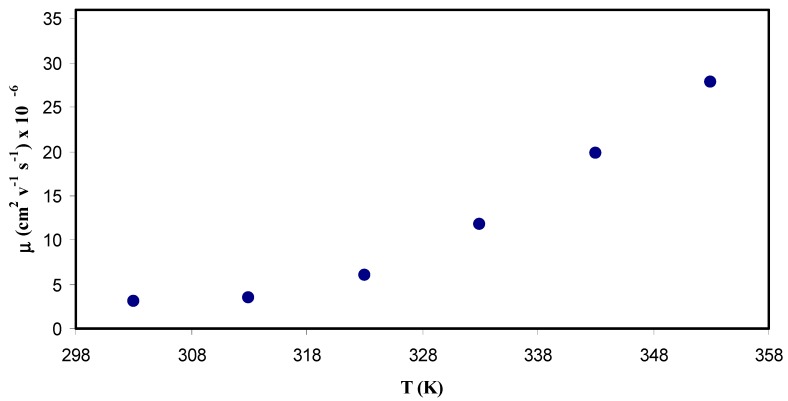
Ion mobility μ as a function of temperature (*T*). It is obvious that the mobility of ions increases nonlinearly with temperature.

Ion mobility can be calculated from the following equation:

(15) $$\mu =\frac{\sigma_{\mathrm{DC}}}{en}$$

where μ is the ionic mobility. Figure 15 shows the temperature and ion mobility relationship. It is seen that the temperature dependence of μ is comparable to that in diffusivity. Indeed, an increase in the mobility resulted from an increase in the temperature, but it is not linear. At a first glance, this increases in the mobility caused by the free volume formation, in which the temperature enhanced free volume in the amorphous phase [77]. The ion mobility was 3 × 10^−6^ cm^2^ V^−1^ s^−1^ at ambient temperature whereas about 3 × 10^−5^ cm^2^ V^−1^ s^−1^ at 353 K. Winie et al. [89] confirmed observation of the ion mobility improvement with an increase in temperature. On the one hand, Majid and Arof [87], obtained the value of mobility (μ) in the range 10^−8^ to 10^−6^ cm^2^ V^−1^s^−1^. Agrawal et al. [90] however, reported 10^−6^ cm^2^ V^−1^ s^−1^ for ionic mobility. On the other hand, Arya and Sharma [83], documented the ion mobility (μ) in the range 10^−10^ to 10^−12^ cm^2^ V^−1^ s^−1^. Recently, Patla et al. [91] reported the ion mobility (μ) in the range 1.8 × 10^−4^ to 9.5 × 10^−11^ cm^2^ V^−1^ s^−1^ for PVDF based polymer nano-composites incorporated with ammonium iodide (NH_4_I) salt.

## 4. Conclusions

In conclusions, Trukhan model has been verified as a promising method for study diffusion coefficient, charge carrier number density and mobility in PVA:LiCF_3_SO_3_ SPE. In this method, peaks in loss tangent spectra were used in the calculations. Using electrical equivalent circuits (EEC), electrical impedance plots (*Z*i vs. *Z*r) are fitted at various temperatures. The results of impedance study demonstrated that the resistivity of the sample decreases with increasing temperature. The decrease of resistance or impedance with increasing temperature distinguished from Bode plots. It has been found that temperature increases lead to lower bulk resistance and increased permittivity. The Argand plots reveal that ion relaxation follows non-Debye model. The position and height of the loss tangent versus frequency plots also change with temperature, with higher temperatures resulting in a positive shift in position and height of the peaks. The dc conductivity showed an Arrhenius type dependence on temperature, so increasing temperature has led to an increase in conductivity; this increase has been explained by the increase in the amorphous phase of the polymer. The diffusion coefficient, obtained from loss tangent plot, also showed a non-linear dependence on temperature, with increases in temperature resulting in an increase in diffusion coefficient. Charge carrier density showed a similar behavior, but the increase with temperature was not very large. Finally, the relation between the mobility and the temperature was seen to be very similar to the one between the diffusion coefficient and the temperature. This shows that the Trukhan model is successful in utilizing complex impedance spectroscopy to analyze ion transport parameters in SPEs.

## Figures and Tables

**Table 1:** The parameters of the circuit elements of the poly (vinyl alcohol) (PVA):LiFC_3_SO_3_ solid electrolyte at various temperatures.

Temperature (K)	p_1_ (rad)	p_2_ (rad)	k_1_ (F^−1^)	k_2_ (F^−1^)	C_1_ (F)	C_2_ (F)
303	0.84	-	5.60 × 10^8^	-	1.79 × 10^−9^	-
323	0.83	-	5.50 × 10^8^	-	1.82 × 10^−9^	-
343	0.75	0.85	8.00 × 10^7^	6.50 × 10^5^	1.25 × 10^−8^	1.56 × 10^−6^
353	0.74	0.80	5.50 × 10^−7^	2.50 × 10^5^	1.82 × 10^−8^	4.00 × 10^−6^

## References

[1] Turner J.A. (1999). A Realizable Renewable Energy Future. Science.

[2] Fergus J.W. (2010). Ceramic and polymeric solid electrolytes for lithium-ion batteries. J. Power Sources.

[3] Ngai K.S., Ramesh S., Ramesh K., Juan J.C. (2016). A review of polymer electrolytes: Fundamental, approaches and applications. Ionics.

[4] Tambelli C., Bloise A., Rosario A., Pereira E., Magon C., Donoso J.P., Pereira E. (2002). Characterisation of PEO–Al2O3 composite polymer electrolytes. Electrochim. Acta.

[5] Xue Z., Heb D., Xie X. (2015). Poly(ethylene oxide)-based electrolytes for lithium-ion batteries. J. Mater. Chem. A.

[6] Hema M., Selvasekerapandian S., Sakunthala A., Arunkumar D., Nithya H. (2008). Structural, vibrational and electrical characterization of PVA–NH4Br polymer electrolyte system. Phys. B Condens. Matter.

[7] Albu A., Maior I., Nicolae C.A., Bocăneală F.L. (2016). Novel PVA proton conducting membranes doped with polyaniline generated by in-situ polymerization. Electrochim. Acta.

[8] Yang C.-C., Hsu S.-T., Chien W.-C. (2005). All solid-state electric double-layer capacitors based on alkaline polyvinyl alcohol polymer electrolytes. J. Power Sources.

[9] Mohamad A.A., Mohamed N.S., Yahya M.Z.A., Othman R., Ramesh S., Alias Y., Arof A.K. (2003). Ionic conductivity studies of poly(vinyl alcohol) alkaline solid polymer electrolyte and its use in nickel–zinc cells. Solid State Ionics.

[10] Polu A.R., Kumar R. (2013). Preparation and characterization of pva based solid polymer electrolytes for electrochemical cell applications. Chin. J. Polym. Sci..

[11] Zhong C., Deng Y., Hu W., Qiao J., Zhang L., Zhang J. (2015). A review of electrolyte materials and compositions for electrochemical supercapacitors. Chem. Soc. Rev..

[12] Horike S., Umeyama D., Kitagawa S. (2013). Ion Conductivity and Transport by Porous Coordination Polymers and Metal–Organic Frameworks. Acc. Chem. Res..

[13] Marcinek M., Syzdek J., Marczewski M., Piszcz M., Niedzicki L., Kalita M., Plewa-Marczewska A., Bitner A., Wieczorek P., Trzeciak T. (2015). Electrolytes for Li-ion transport—Review. Solid State Ionics.

[14] Siekierski M., Wieczorek W., Przyłuski J. (1998). AC conductivity studies of composite polymeric electrolytes. Electrochim. Acta.

[15] Bandara L.R.A.K., Dissanayake M.A.K.L., Mellander B.-E. (1998). Ionic conductivity of plasticized (PEO)-LiCF3SO3 electrolytes. Electrochim. Acta.

[16] Klein R.J., Zhang S., Dou S., Jones B.H., Colby R.H., Runt J. (2006). Modeling electrode polarization in dielectric spectroscopy: Ion mobility and mobile ion concentration of single-ion polymer electrolytes. J. Chem. Phys..

[17] Bhattacharja S., Smoot S.W., Whitmore D.H. (1986). Cation and anion diffusion in the amorphous phase of the polymer electrolyte (PEO) 8LiCF3SO3. Solid State Ionics..

[18] Every H.A., Zhou F., Forsyth M., MacFarlane D.R. (1998). Lithium ion mobility in poly (vinyl alcohol) based polymer electrolytes as determined by 7Li NMR spectroscopy. Electrochim. Acta.

[19] Saroj A., Singh R. (2012). Thermal, dielectric and conductivity studies on PVA/Ionic liquid [EMIM][EtSO4] based polymer electrolytes. J. Phys. Chem. Solids..

[20] Hema M., Selvasekarapandian S., Hirankumar G., Sakunthala A., Arunkumar D., Nithya H. (2010). Laser Raman and ac impedance spectroscopic studies of PVA: NH4NO3 polymer electrolyte. Spectrochim. Acta Part A Mol. Biomol. Spectrosc..

[21] Johansson A., Wendsjö Å., Tegenfeldt J. (1992). NMR spectroscopy of peo-based polymer electrolytes. Electrochim. Acta.

[22] Arof A.K., Amirudin S., Yusof S.Z., Noor I.M. (2014). A method based on impedance spectroscopy to determine transport properties of polymer electrolytes. Phys. Chem. Chem. Phys..

[23] Munar A., Andrio A., Iserte R., Compañ V. (2011). Ionic conductivity and diffusion coefficients of lithium salt polymer electrolytes measured with dielectric spectroscopy. J. Non-Cryst. Solids.

[24] Aziz S.B., Abidin Z.H.Z. (2015). Ion-transport study in nanocomposite solid polymer electrolytes based on chitosan: Electrical and dielectric analysis. J. Appl. Polym. Sci..

[25] Aziz S.B., Abidin Z.H.Z. (2014). Electrical and morphological analysis of chitosan:AgTf solid electrolyte. Mater. Chem. Phys..

[26] Aziz S.B., Mamand S.M. (2018). The Study of Dielectric Properties and Conductivity Relaxation of Ion Conducting Chitosan: NaTf Based Solid Electrolyte. Int. J. Electrochem. Sci..

[27] Aziz S.B., Brza M.A., Kadir M.F.Z., Hamsan M.H., Abidin Z.H.Z., Tahir D.A., Abdullah O.G. (2019). Investigation on Degradation and Viscoelastic Relaxation of Li Ion in Chitosan Based Solid Electrolyte. Int. J. Electrochem. Sci..

[28] Aziz S.B. (2013). Li+ ion conduction mechanism in poly (ε-caprolactone)-based polymer electrolyte. Iran Polym.J..

[29] Hatchett D.W., Josowicz M. (2008). Composites of Intrinsically Conducting Polymers as Sensing Nanomaterials. Chem. Rev..

[30] Cho S., Chen C.-F., Mukherjee P.P. (2015). Influence of Microstructure on Impedance Response in Intercalation Electrodes. J. Electrochem. Soc..

[31] Svensson A.M., Valøen L.O., Tunold R. (2005). Modeling of the impedance response of porous metal hydride electrodes. Electrochim. Acta.

[32] Kumar M., Tiwari T., Chauhan J.K., Srivastava N. (2014). Understanding the ion dynamics and relaxation behavior from impedance spectroscopy of NaI doped Zwitterionic polymer system. Mater. Res. Express.

[33] Aziz S., Abidin Z.H.Z., Arof A.K. (2010). Influence of silver ion reduction on electrical modulus parameters of solid polymer electrolyte based on chitosan-silver triflate electrolyte membrane. Express Polym. Lett..

[34] Fu K.K., Gong Y., Dai J., Gong A., Han X., Yao Y., Wang C., Wang Y., Chen Y., Yan C. (2016). Flexible, solid-state, ion-conducting membrane with 3D garnet nanofiber networks for lithium batteries. Proc. Natl. Acad. Sci. USA.

[35] Malathi J., Kumaravadivel M., Brahmanandhan G.M., Hema M., Baskaran R., Selvasekarapandian S. (2010). Structural, thermal and electrical properties of PVA–LiCF3SO3 polymer electrolyte. J. Non-Cryst. Solids.

[36] Aziz S.B., Brza M., Mohamed P.A., Kadir M., Hamsan M., Abdulwahid R.T., Woo H. (2019). Increase of metallic silver nanoparticles in Chitosan:AgNt based polymer electrolytes incorporated with alumina filler. Results Phys..

[37] Pradhan D.K., Choudhary P., Samantaray B.K., Karan N.K., Katiyar R.S. (2007). Effect of Plasticizer on Structural and Electrical Properties of Polymer Nanocompsoite Electrolytes. Int. J. Electrochem. Sci..

[38] Mohapatra S.R., Thakur A.K., Choudhary R.N.P. (2009). Effect of nanoscopic confinement on improvement in ion conduction and stability properties of an intercalated polymer nanocomposite electrolyte for energy storage applications. J. Power Sources.

[39] Aziz S.B., Abdullah R.M., Kadir M., Ahmed H.M. (2019). Non suitability of silver ion conducting polymer electrolytes based on chitosan mediated by barium titanate (BaTiO_3_) for electrochemical device applications. Electrochim. Acta.

[40] Shukur M., Ithnin R., Kadir M. (2014). Electrical characterization of corn starch-LiOAc electrolytes and application in electrochemical double layer capacitor. Electrochim. Acta.

[41] Teo L.P., Buraidah M.H., Nor A.F.M., Majid S.R. (2012). Conductivity and dielectric studies of Li2SnO3. Ionics.

[42] Eftekhari A. (2018). The mechanism of ultrafast supercapacitors. J. Mater. Chem. A.

[43] Aziz S.B., Faraj M.G., Abdullah O.G. (2018). Impedance Spectroscopy as a Novel Approach to Probe the Phase Transition and Microstructures Existing in CS:PEO Based Blend Electrolytes. Sci. Rep..

[44] Cebeci F.Ç., Geyik H., Sezer E., Sarac A.S. (2007). Synthesis, electrochemical characterization and impedance studies on novel thiophene-nonylbithiazole-thiophenecomonomer. J. Electroanal. Chem..

[45] Vergaz R., Barrios D., Sánchez-Pena J.-M., Pozo-Gonzalo C., Salsamendi M. (2009). Relating cyclic voltammetry and impedance analysis in a viologenelectrochromic device. Sol. Energy Mater. Sol. Cells.

[46] Aziz S.B., Hamsan M.H., Abdullah R.M., Kadir M.F.Z. (2019). A Promising Polymer Blend Electrolytes Based on Chitosan: Methyl Cellulose for EDLC Application with High Specific Capacitance and Energy Density. Molecules.

[47] Vijil Vani C., Thanikaikarasan S., Mahalingam T., Sebastian P.J., Verea L.E., Shajan X.S. (2014). Effect of X-ray Irradiation on Dielectric Properties of Polymer Electrolytes Complexed with LiCF_3_SO_3_. J. New Mater. Electrochem. Syst..

[48] Pradhan D.K., Choudhary R.N.P., Samantaray B.K. (2008). Studies of Dielectric Relaxation and AC Conductivity Behavior of Plasticized Polymer Nanocomposite Electrolytes. Int. J. Electrochem. Sci..

[49] Woo H.J., Majid S.R., Arof A.K. (2012). Dielectric properties and morphology of polymer electrolyte based on poly(ε-caprolactone) and ammonium thiocyanate. Mater. Chem. Phys..

[50] Ravi M., Pavani Y., Kumar K.K., Bhavani S., Sharma A., Rao V.N. (2011). Studies on electrical and dielectric properties of PVP:KBrO_4_ complexed polymer electrolyte films. Mater. Chem. Phys..

[51] Polu A.R., Kumar R. (2011). AC impedance and dielectric spectroscopic studies of Mg^2+^ ion-conducting PVA–PEG blended polymer electrolytes. Bull. Mater. Sci..

[52] Das-Gupta D. (2001). Molecular processes in polymer electrets. J. Electrost..

[53] Kumar M.S., Bhat D.K. (2009). Polyvinyl alcohol–polystyrene sulphonic acid blend electrolyte for supercapacitor application. Phys. B.

[54] Pradhan D.K., Choudhary R.N.P., Samantaray B.K. (2009). Studies of dielectric and electrical properties of plasticized polymer nanocompositeelectrolytes. Mater. Chem. Phys..

[55] Tian F., Ohki Y. (2014). Electric modulus powerful tool for analyzing dielectric behavior. IEEE Trans. Dielectr. Electr. Insul..

[56] Aziz S.B., Woo T.J., Kadir M.F., Ahmed H.M., Ahmed H.M. (2018). A conceptual review on polymer electrolytes and ion transport models. J. Sci. Adv. Mater. Devices.

[57] Aziz S.B. (2016). Occurrence of electrical percolation threshold and observation of phase transition in chitosan(1 − x):AgIx (0.05 ≤ x ≤ 0.2)-based ion-conducting solid polymer composites. Appl. Phys. A.

[58] Aziz S.B. (2016). Role of Dielectric Constant on Ion Transport: Reformulated Arrhenius Equation. Adv. Mater. Sci. Eng..

[59] Aziz S.B. (2018). The Mixed Contribution of Ionic and Electronic Carriers to Conductivity in Chitosan Based Solid Electrolytes Mediated by CuNt Salt. J. Inorg. Organomet. Polym. Mater..

[60] Aziz S.B., Abdullah R.M. (2018). Crystalline and amorphous phase identification from the tanδ relaxation peaks and impedance plots in polymer blend electrolytes based on [CS:AgNt]x:PEO(x−1) (10 ≤ x ≤ 50). Electrochim. Acta.

[61] Agrawal S.L., Singh M., Tripathi M., Dwivedi M.M., Pandey K. (2009). Dielectric relaxation studies on [PEO–SiO_2_]:NH4SCN nanocomposite polymer electrolyte films. J. Mater. Sci..

[62] Karmakar A., Ghosh A. (2012). Dielectric permittivity and electric modulus of polyethylene oxide (PEO)–LiClO4 composite electrolytes. Curr. Appl. Phys..

[63] Sengwa R.J., Choudhary S., Sankhla S. (2008). Low frequency dielectric relaxation processes and ionic conductivity of montmorillonite clay nanoparticles colloidal suspension in poly(vinyl pyrrolidone)−ethylene glycol blends. Express Polym. Lett..

[64] Castillo J., Chacon M., Castillo R., Vargas R.A., Bueno P.R., Varela J.A. (2009). Dielectricrelaxation and dcconductivityonthe PVOH-CF3COONH4 polymersystem. Ionics.

[65] Bello A., Laredo E., Grimau M. (2007). Comparison of analysis of dielectric spectra of PCL in the ε∗ and the M∗ formalism. J. Non-Cryst. Solids.

[66] Reddy C.V.S., Han X., Zhu Q.-Y., Mai L.-Q., Chen W. (2006). Dielectric spectroscopy studies on (PVP + PVA) polyblend film. Microelectron. Eng..

[67] Yamamoto T., Inami M., Kanbara T. (1994). Preparation and properties of polymer solid electrolytes using poly(vinyl alcohol) and thermally resistive poly[arylene(1,3-imidazolidine-2,4,5-trione-1,3-diyl)] as matrix polymers. Chem. Mater..

[68] Takaki K., Minoru I., Takakazu Y., Atsushi N., Tooru T., Masayoshi W., Naoya O. (1989). New Lithium Salt Ionic Conductor Using Poly(vinyl alcohol) Matrix. Chem. Lett..

[69] Sheha E., Khoder H., Shanap T.S., El-Shaarawy M.G., El Mansy M.K. (2012). Structure, dielectric and optical properties of p-type (PVA/CuI) nanocomposite polymer electrolyte for photovoltaic cells. Optik.

[70] Choi U.H., Liang S., Chen Q., Runt J., Colby R.H. (2016). Segmental Dynamics and Dielectric Constant of Polysiloxane Polar Copolymers as Plasticizers for Polymer Electrolytes. ACS Appl. Mater. Interfaces.

[71] Aziz S.B. (2015). Study of electrical percolation phenomenon from the dielectric and electric modulus analysis. Bull. Mater. Sci..

[72] Aziz S.B., Abdullah R.M., Rasheed M.A., Ahmed H.M. (2017). Role of Ion Dissociation on DC Conductivity and Silver Nanoparticle Formation in PVA:AgNt Based Polymer Electrolytes: Deep Insights to Ion Transport Mechanism. Polymers.

[73] Mohan V.M., Qiu W., Shen J., Chen W. (2010). Electrical properties of poly(vinyl alcohol) (PVA) based on LiFePO4 complex polymer electrolyte films. J. Polym. Res..

[74] Fan L., Dang Z., Wei G., Nan C.W., Li M. (2003). Effect of nanosizedZnO on the electrical properties of (PEO)16LiClO4 electrolytes. Mater. Sci. Eng. B.

[75] Jayathilaka P.A.R.D., Dissanayake M.A.K.L., Albinsson I., Mellander B.E. (2003). Dielectric relaxation, ionic conductivity and thermal studies of the gel polymer electrolyte system PAN/EC/PC/LiTFSI. Solid State Ionics.

[76] Khatri P., Behera B., Srinivas V., Choudhary R.N.P. (2009). Structural and dielectric properties of Ba3V2O8 ceramics. Curr. Appl. Phys..

[77] Louati B., Hlel F., Guidara K. (2009). Ac electrical properties and dielectric relaxation of the new mixed crystal (Na0.8Ag0.2)2PbP2O7. J. Alloy. Compd..

[78] Idris N.H., Senin H.B., Arof A.K. (2007). Dielectric spectra of LiTFSI-doped chitosan/PEO blends. Ionics.

[79] Fan F. (2015). Ion Transport in Polymer Electrolytes. Ph.D. Thesis.

[80] Tiong T.S., Buraidah M.H., Teo L.P., Arof A.K. (2016). Conductivity studies of poly(ethylene oxide)(PEO)/poly(vinyl alcohol) (PVA) blend gel polymer electrolytes for dye-sensitized solar cells. Ionics.

[81] Eschen T., Kösters J., Schönhoff M., Stolwijk N.A. (2012). Ionic Transport in Polymer Electrolytes Based on PEO and the PMImI Ionic Liquid: Effects of Salt Concentration and Iodine Addition. J. Phys. Chem. B.

[82] Aziz S.B., Abidin Z., Arof A. (2010). Effect of silver nanoparticles on the DC conductivity in chitosan–silver triflate polymer electrolyte. Phys. B Condens. Matter.

[83] Arya A., Sharma A.L. (2018). Optimization of salt concentration and explanation of two peakpercolation in blend solid polymer nanocomposite films. J. Solid State Electrochem..

[84] Sun B., Mindemark J., Morozov E.V., Costa L.T., Bergman M., Johansson P., Fang Y., Furó I., Brandell D. (2016). Ion transport in polycarbonate based solid polymer electrolytes: Experimental and computational investigations. Phys. Chem. Chem. Phys..

[85] Rice M.J., Roth W.L. (1972). Ionic transport in super ionic conductors: A theoretical model. J. Solid State Chem..

[86] Maurya K.K., Hashmi S.A., Chandra S., Chowdari B.V.R., Chandra S., Singh S., Srivastava P.C. (1992). Evidence of ion association in polymer electrolyte by direct mobility measurement. Solid State Ionics: Materials and Applications.

[87] Majid S., Arof A. (2005). Proton-conducting polymer electrolyte films based on chitosan acetate complexed with NH4NO3 salt. Phys. B Condens. Matter.

[88] Chandra A., Agrawal R.C., Mahipal Y.K. (2009). Ion transport property studies on PEO–PVP blended solid polymer electrolyte membranes. J. Phys. D Appl. Phys..

[89] Winie T., Ramesh S., Arof A. (2009). Studies on the structure and transport properties of hexanoyl chitosan-based polymer electrolytes. Phys. B Condens. Matter.

[90] Agrawal R.C., Chandra A., Bhatt A., Mahipal Y.K. (2008). Investigations on ion transport properties of and battery discharge characteristic studies on hot-pressed Ag+-ion-conducting nano-composite polymer electrolytes: (1 − x) [90PEO:10AgNO_3_]:xSiO_2_. New J. Phys..

[91] Patla S.K., Ray R., Karmakar S., Das S., Tarafdar S. (2019). Nanofiller-Induced Ionic Conductivity Enhancement and Relaxation Property Analysis of the Blend Polymer Electrolyte Using Non-Debye Electric Field Relaxation Function. J. Phys. Chem. C.

